# Exploring the tissue tropism of pseudorabies virus based on miRNA level analysis

**DOI:** 10.1186/s12866-019-1497-4

**Published:** 2019-06-11

**Authors:** Yi Fan, Ling Zhu, Xiangang Sun, Wenting Lyu, Lei Xu, Yue Yin, Jun Zhao, Jianbo Huang, Yichao Den, Zhiyi Jiang, Shiyao Xu, Xiyu Mao, Zhiwen Xu

**Affiliations:** 1 Present Address: College of Veterinary Medicine, Sichuan Agricultural University, Huimin Road 211, weenjiang district, Chengdu, Sichuan China; 2 Key Laboratory of Animal Diseases and Human Health of Sichuan Province, Chengdu, Sichuan China

**Keywords:** Pseudorabies virus, miRNA, Olfactory bulb, Lung, Spleen, Tissue tropism

## Abstract

**Background:**

Pseudorabies virus (PRV, or suid herpesvirus, SuHV-1), a member of the herpesvirus family, has an extremely broad host range and threatens the pig industry in China. PRV can evade host innate immunity and infect the kidney, lung, brain and other tissues. At the same time, many studies have reported that microRNA (miRNA) can affect the replication of viruses by regulating gene expression levels.

**Results:**

Here, to identify changes in miRNA expression and post-transcriptional regulation associated with PRV infection in the lung, spleen, and olfactory bulb, we sequenced small RNAs in tissues of rats infected or uninfected with PRV strain XJ (PRV-XJ). Sixty-one, 199 and 29 differentially-expressed miRNAs were identified in the lung, spleen, and olfactory bulb, respectively, of infected compared with uninfected rats. Among the miRNAs differentially-expressed in PRV-infected rats, 36, 171, and 15 miRNAs showed tissue-selective expression in the olfactory bulb, lung and spleen, respectively. All differentially-expressed miRNAs were analyzed for their GO functional annotations and KEGG pathway associations .

**Conclusions:**

In PRV-XJ-infected rats, miRNAs were differentially expressed in the lung, spleen and olfactory bulb. These miRNAs were involved in regulating various pathways of the nervous, respiratory and immune systems, and may affect the tissue tropism of the virus and play pivotal roles in viral infection and proliferation.

## Background

Pseudorabies virus (PRV, or suid herpesvirus, SuHV-1; herpesvirus family, alphaherpesvirus subfamily) shows the typical virion structure of the alphaherpesviruses. Pigs are the natural hosts of PRV and the source of most human infections [1]. The virus is spread by horizontal propagation, through food, water and feces. PRV can infect primary sensory neurons. After proliferating in primary sensory neurons, transmission can occur trans-synaptically to second-order neurons [2, 3].

PRV infection can result in respiratory diseases, such as hemorrhagic rhinitis and pharyngitis. PRV-infected pigs can develop interstitial pneumonia, hemorrhagic pneumonia, and significantly increasing widths of the alveolar septum and the intralobular interstitium. PRV-infected fetuses sometimes show hemorrhagic necrosis. In piglets, PRV infection can cause pulmonary edema, bleeding, and lobular pneumonia, and occasionally results in gray necrosis on the lung surface. PRV can evade host anti-viral immunity in two major ways. First, PRV escapes humoral immunity by interfering with complement regulatory pathways: for example, PRV glycoprotein C can bind porcine C3, and other viral proteins can block the binding of chemokines to their receptors. Second, PRV escapes cellular immunity: for example, PRV glycoprotein D can regulate CD112 [4], thus inhibiting NK cell-mediated degranulation and lysis, and other viral proteins can inhibit antigen presentation and expression of interferon-regulatory genes [5].

MicroRNAs (miRNAs) are recently-discovered small endogenous non-coding RNA molecules, and are only 21–25 nucleotides in length [6, 7]. They are widely found in eukaryotes and serve to repress the expression of target genes through degradation of mRNAs at the transcriptional level after miRNA binding to 3’UTRs. MiRNAs, which account for about 2% of genomic sequences, are widespread in multicellular organisms [8]. They have been highly conserved over biological evolution and regulate a variety of physiological processes through involvement in development, apoptosis, fat metabolism, neuronal development, cell differentiation, and hormone secretion [9, 10]. MicroRNAs are essential regulators of central nervous system development and also play an important role in stabilizing cell differentiation [11, 12] .Previous research has reported alterations of miRNA expression during PRV infection.

In this study, we infected rats with PRV-XJ strain via intramuscular injection in the thigh. The miRNA content of various tissues was determined by high-throughput sequencing to identify differentially-expressed miRNAs. Our overall goals were to study the regulatory effects of miRNA on PRV infection and proliferation, to understand the tissue tropism of PRV, and to clarify the roles of miRNAs in regulating host antiviral responses.

## Results

### Solexa sequencing analysis

Using Bowtie software, filtered reads were compared with the Silva, GtRNAdb, Rfam and Repbase databases to filter out rRNA, tRNA, snRNA, RNA (snoRNA) and other ncRNA and repeat sequences, yielding unannotated reads (Table 1). The unannotated reads were compared with the reference genome (rattus_norvegicus: ftp://ftp.ensembl.org/pub/release-85/fasta/rattus_norvegicus/) to obtain positional information (mapped reads) (Table 2).

**Table 1:** Distribution of sRNAs coverage in PRV-infected and uninfected rat tissues

Libraries		Total	rRNA	snRNA	snoRNA	tRNA	Repbase	Unannotated
PRV-Olf	Number	17,351,169	227,202	134	53,917	72,211	132,963	16,864,742
	(%)	100.00	1.31	0.00	0.31	0.42	0.77	97.19
PRV-Lung	Number	23,653,337	1,004,480	44	115,666	142,674	207,449	22,183,024
	(%)	100.00	4.25	0.00	0.49	0.60	0.88	93.78
PRV-Spleen	Number	19,818,550	2,821,203	240	178,427	1,849,425	444,541	14,524,714
	(%)	100.00	14.24	0.00	0.90	9.33	2.24	73.29
Olf	Number	17,715,320	414,799	75	61,604	259,351	128,916	16,850,575
	(%)	100.00	2.34	0.00	0.35	1.46	0.73	95.12
Lung	Number	21,327,335	1,542,635	52	107,676	73,396	399,711	19,203,865
	(%)	100.00	7.23	0.00	0.50	0.34	1.87	90.06
Spleen	Number	17,006,737	4,374,703	130	334,357	101,551	541,772	11,654,224
	(%)	100.00	25.72	0.00	1.97	0.60	3.19	68.52

**Table 2:** sRNA mapped reads in PRV-infected and uninfected rat tissues

Libraries	Unannotated	Mapped Reads	Mapped Reads(+)	Mapped Reads(−)
PRV-Olf	16,864,742	11,872,944	4,755,973	7,116,971
PRV-Lung	22,183,024	15,005,824	9,870,656	5,135,168
PRV-Spleen	14,524,714	8,986,565	4,720,073	4,266,492
Olf	16,850,575	11,665,256	5,357,448	6,307,808
Lung	19,203,865	12,165,741	7,797,023	4,368,718
Spleen	11,654,224	7,732,240	4,039,038	3,693,202

**Fig. 1:**
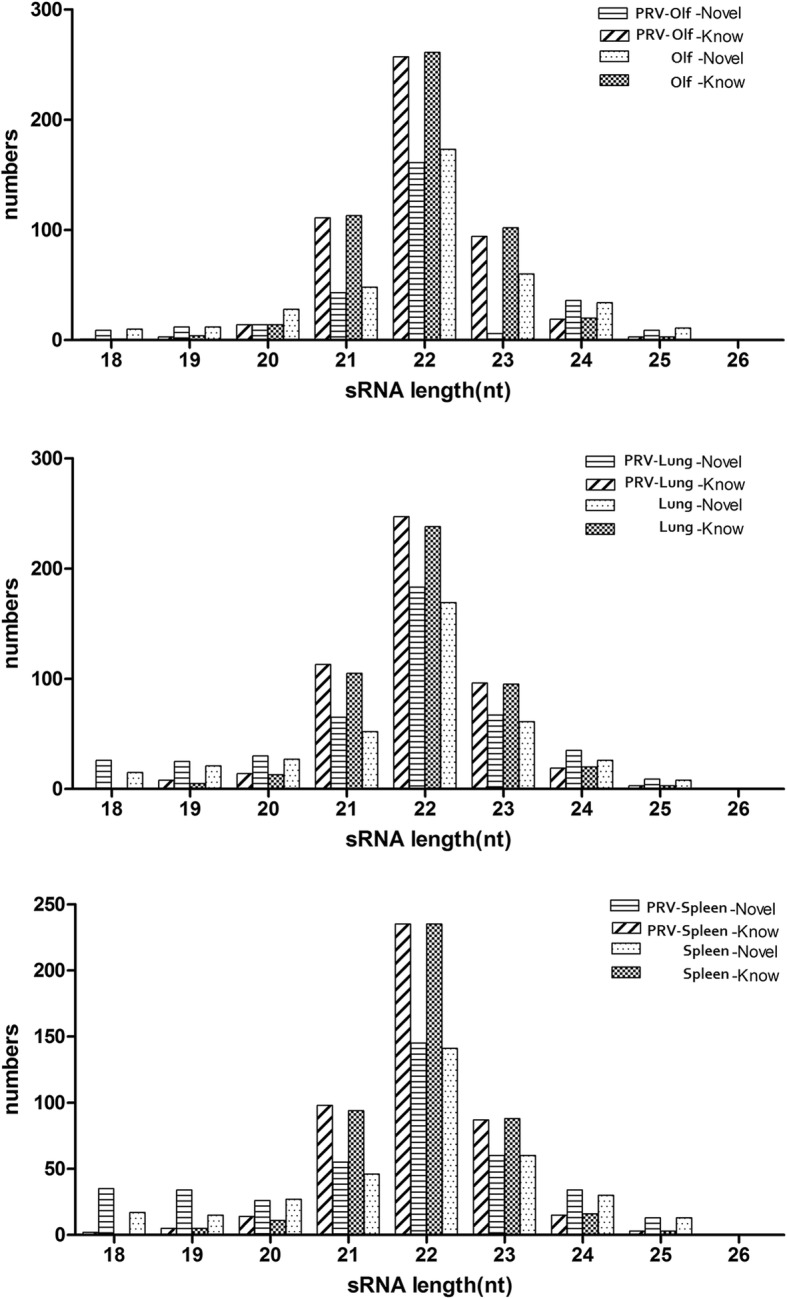
Length distribution of previously-described miRNAs and novel miRNAs (18–26 nt) in tissues of PRV-infected and uninfected rats

Previously-described and novel miRNAs were compared with reference genome sequences and identified using miRDeep2 software (Table 3). Due to the specificity of the Dicer and DCL enzymes, the lengths of mature miRNAs are primarily in the range of 20–24 nt. The length distributions of identified miRNAs and novel unidentified miRNAs are shown in Fig. 1.

**Table 3:** Previously-described and novel miRNAs in tissues of PRV-infected and uninfected rats

Libraries	Known-miRNAs	novel-miRNAs	Total
PRV-Olf	502	345	847
PRV-Lung	500	440	940
PRV-Spleen	459	402	861
Olf	518	376	894
Lung	479	379	858
Spleen	452	349	801
Total	595	688	1283

### MiRNA RT-qPCR assay

**Fig. 2:**
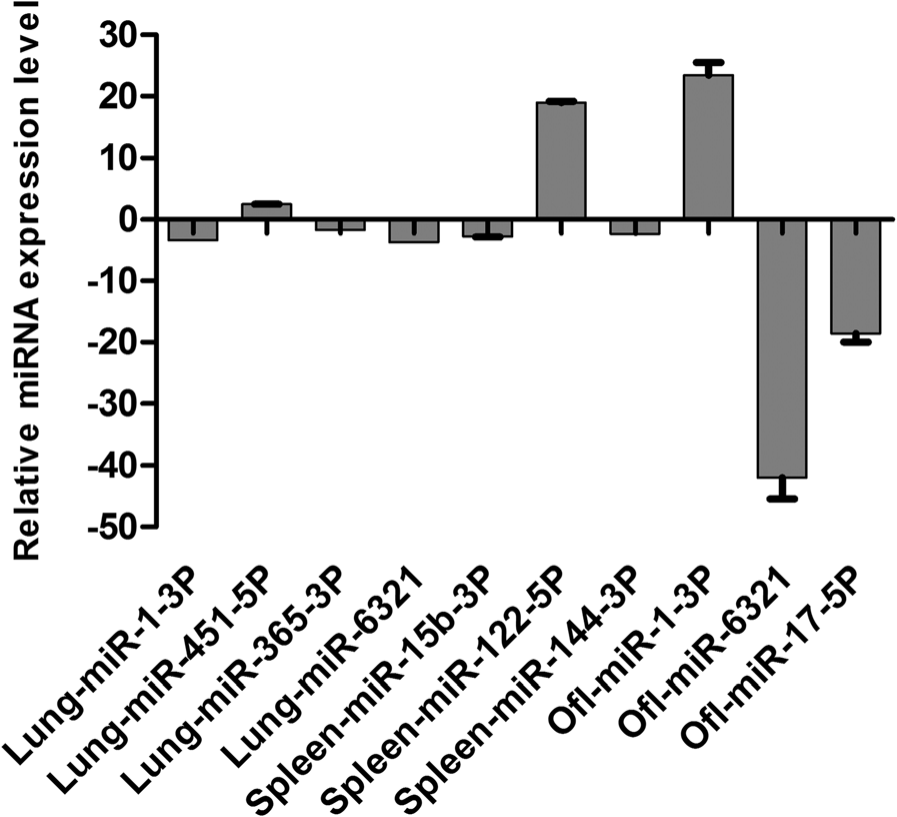
Validation of differentially-expressed host miRNAs using polyA RT-qPCR. The experiments were performed with at least three independent replicates

MiRNA RT-qPCR was used to verify the expression levels of host rat-encoded candidate miRNAs to validate the deep sequencing results. Ten differentially-expressed miRNAs in infected rats were randomly selected and quantified using this approach; non-conserved miRNAs were excluded. Each group has three repetitions (Fig. 2). Overall, the relative miRNA expression levels determined using deep sequencing were consistent with the levels of these miRNAs quantified using RT-qPCR.

### Identification of differentially-expressed miRNAs

**Fig. 3:**
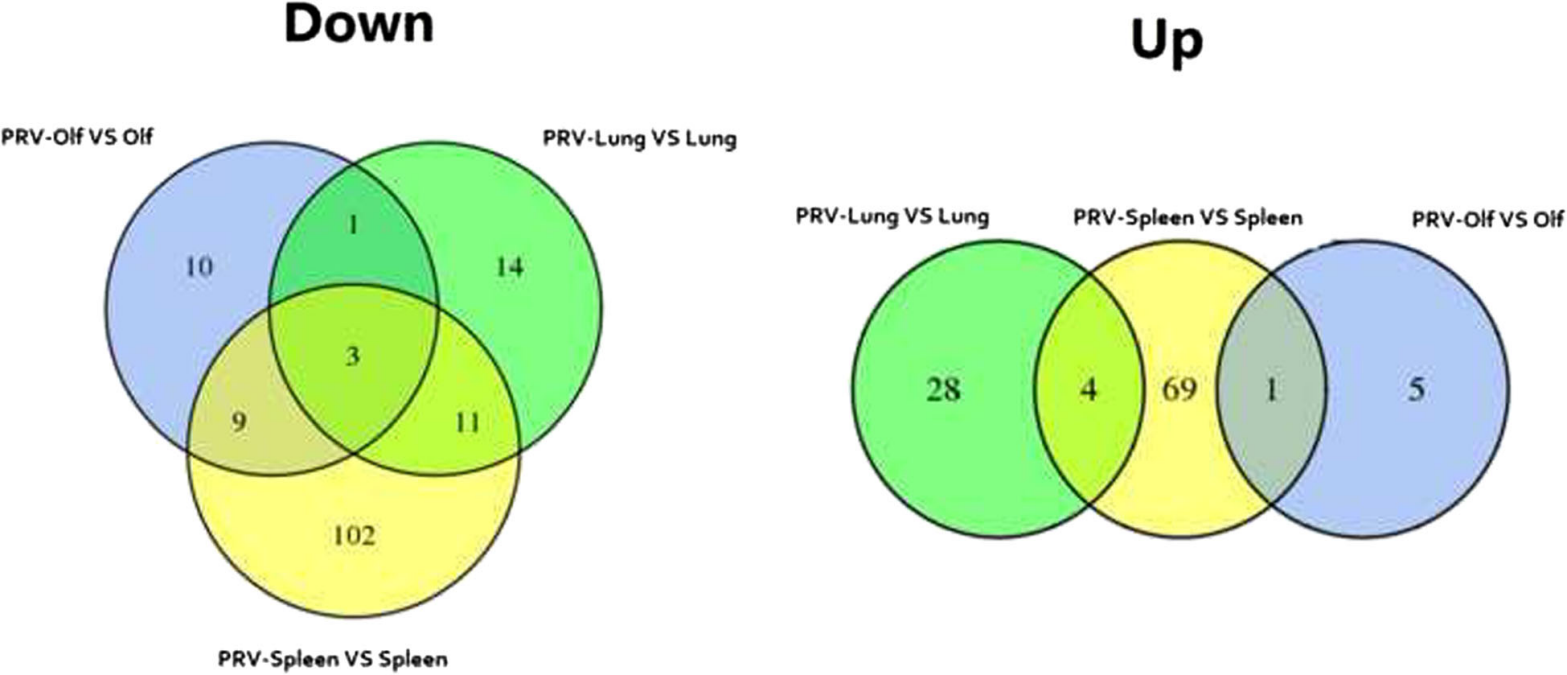
Comparison of differentially-expressed miRNAs that are down-regulated (left) or up-regulated (right) with PRV infection in each tissue

**Fig. 4:**
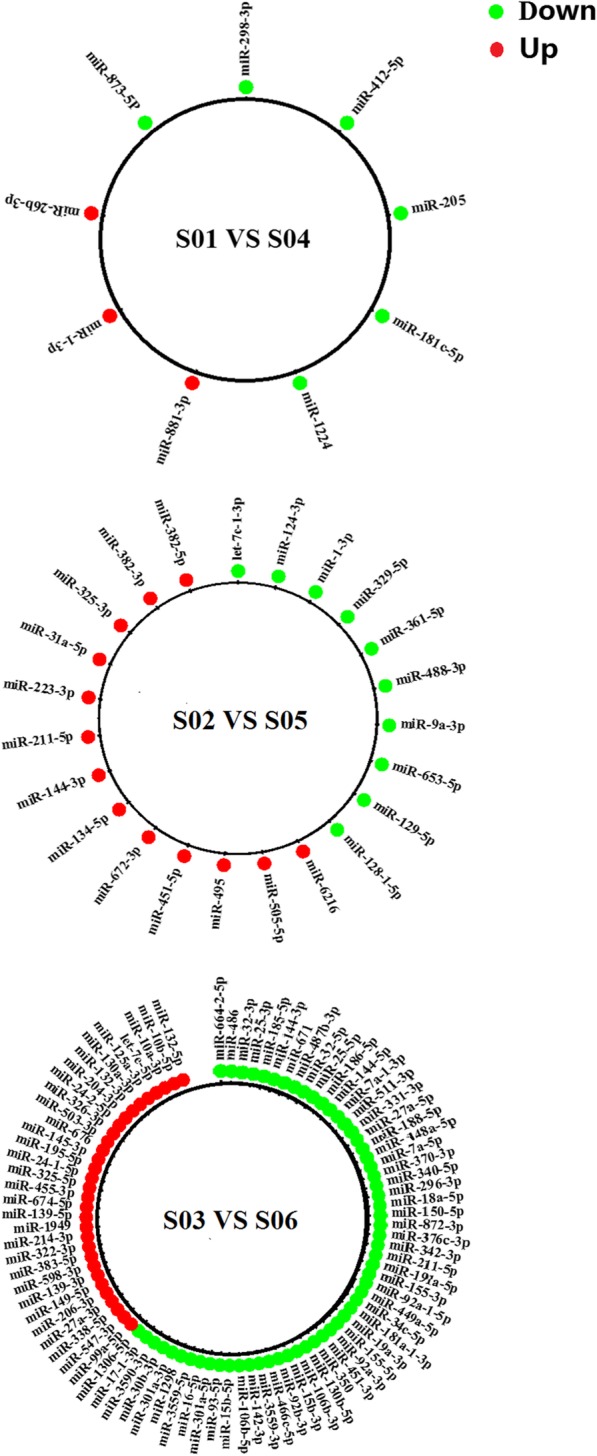
MiRNAs with tissue-specific differential expression in response to PRV infection. Non-conserved miRNAs have been excluded. Red bars (right) represent up-regulated miRNAs and green bars (left) represent down-regulated miRNAs, ordered from most strongly up-regulated (top) to most strongly down-regulated (bottom)

Levels of miRNA in various tissues were compared in infected and uninfected rats by analyzing the Solexa sequencing results. Differentially-expressed miRNAs between infected and uninfected samples were identified using IDEG6 [13] software using | log2 (FC) | > = 1 and FDR < = 0.01 as screening criteria. Fold change (FC) represents the ratio of miRNA expression between two samples. *P*-values can be interpreted as the probability of there being no true difference in miRNA expression given the experimental data. Finally, false discovery rate (FDR) was used as a reference indicator of differential miRNA expression. The number of differentially-expressed miRNAs in infected vs. uninfected rats is shown in Fig. 3. Tissue-specific differentially-expressed miRNAs were identified by comparing the sets of differentially-expressed miRNAs in each tissue (Fig. 4).

Twenty-nine differentially-expressed miRNAs were identified by comparing miRNA sequences from the olfactory bulbs of infected and uninfected rats, of which six were non-conserved miRNAs (These miRNAs have different functions in different species) and 23 were conserved miRNAs. Excluding the six non-conserved miRNAs, four conserved miRNAs were up-regulated and 19 were down-regulated in infected rats. Fifteen differentially-expressed miRNAs were identified with enriched expression in olfactory bulbs, including six down-regulated miRNAs, three up-regulated miRNAs and six non-conserved miRNAs.

Sixty-one differentially-expressed miRNAs were identified in the lungs of infected rats. Forty-two differentially-expressed miRNAs were identified with enriched expression in the lung, of which 10 were down-regulated miRNAs, 13 were up-regulated miRNAs and 19were non-conserved miRNAs.

In total, 199 differentially-expressed miRNAs were identified in the spleens of infected compared with uninfected rats. Moreover, 171differentially-expressed miRNAs were identified with enriched expression in the spleen, of which 58 were down-regulated miRNAs, 31 were up-regulated miRNAs and 82 were non-conserved miRNAs.

**Fig. 5:**
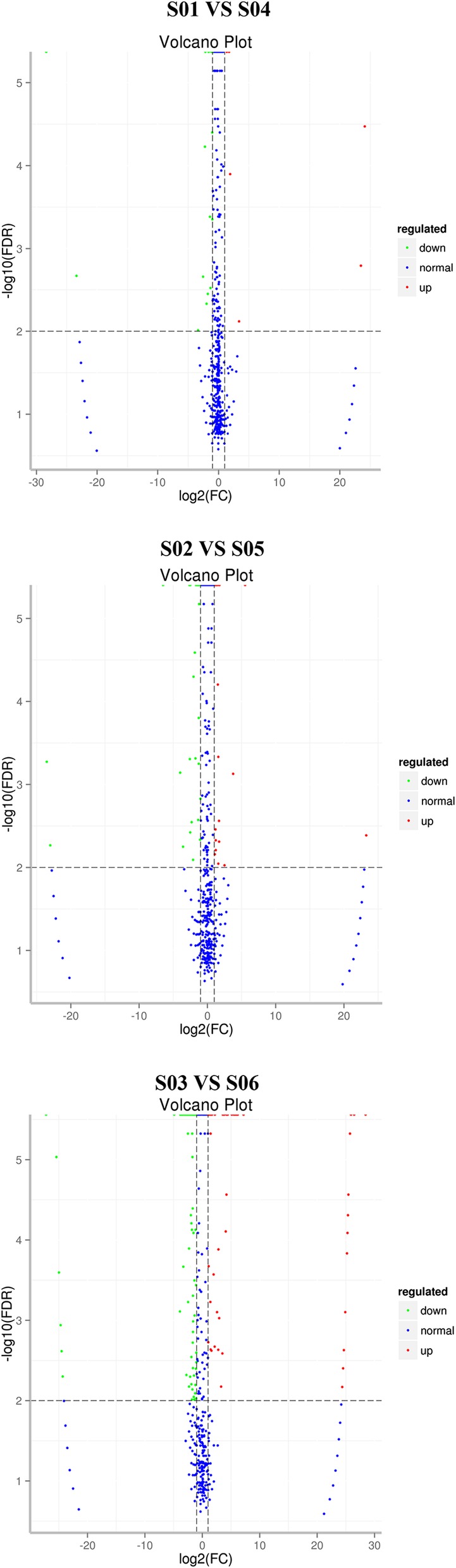
Volcano plot of differentially-expressed miRNAs. Each point represents a miRNA; the x-axis represents the logarithm of the fold difference in miRNA expression between the two tissues; and the y-axis represents the negative logarithm of the false discovery rate. The blue points represent non-differentially expressed miRNAs, the red points represent up-regulated miRNAs, and the green points represent down-regulated miRNAs

**Fig. 6:**
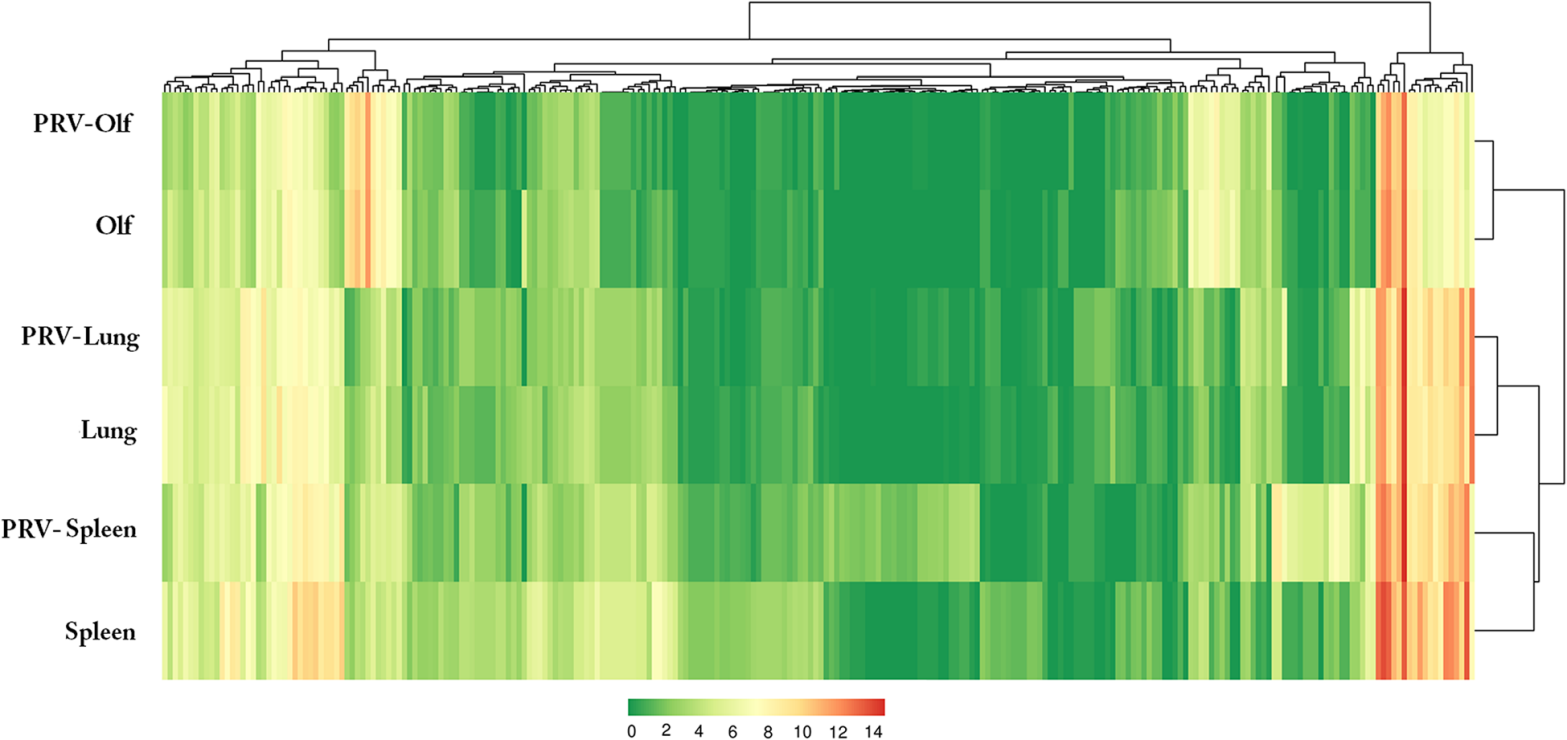
Clustering of differentially-expressed miRNAs. Columns represent different tissues; row represent different miRNAs. The cluster analysis used the log 10 (TPM + 1) values; red indicates high expression of miRNA and green indicates low expression of miRNA

Differences in miRNA expression levels between two samples, as well as the statistical significance of these differences, can be seen in the volcano plot in Fig. 5. A cluster analysis was used to assess whether differentially-expressed miRNAs had the same or similar expression patterns (Fig. 6).

### Gene annotations of differentially-expressed miRNAs

MiRnada(v3.3a) [14] and RNAhybrid(v2.1.1) [15] software were used to predict the target genes of differentially-expressed miRNAs. The predicted target gene sequences were compared with the NR [16], COG [17], GO [18], KEGG [19], SWISS-PROT [20], KOG [21], and Pfam [22] databases using BLAST(v2.2.26) software to obtain target gene annotation information (Table 4).

**Table 4:** Target gene annotations of differentially-expressed miRNAs

Type	COG	GO	KEGG	KOG	Pfam	Swiss-Prot	eggNOG	NR
PRV-Olf vs Olf	544	1513	1054	1053	1584	1640	1661	1677
PRV-Lung vs Lung	354	1072	705	713	1094	1146	1163	1172
PRV-Spleen vs Spleen	1869	5385	3685	3575	5609	5843	5937	5999

### GO annotations and KEGG pathway analysis of target genes of differentially-expressed miRNAs

**Fig. 7:**
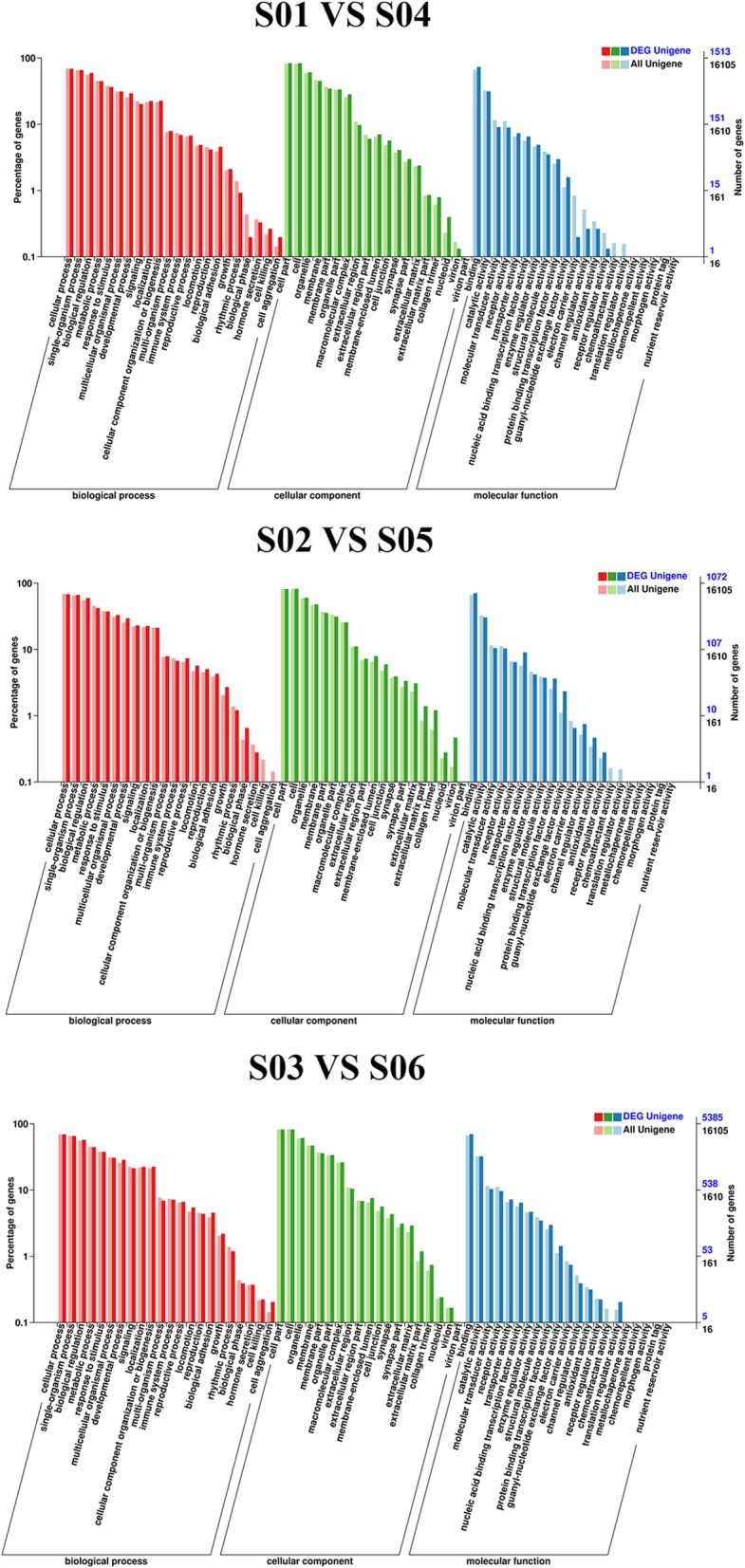
GO classification statistics of differentially-expressed miRNA target genes. The abscissa shows GO classification, the left vertical axis shows the percentage of the total number of genes, and the right vertical axis shows the number of genes. This figure shows the gene enrichment of GO secondary functions in differentially-expressed miRNA target genes and in all genes. The obvious difference in proportion of secondary functions shows that miRNA target genes and all genes have different enrichment tendencies

**Fig. 8:**
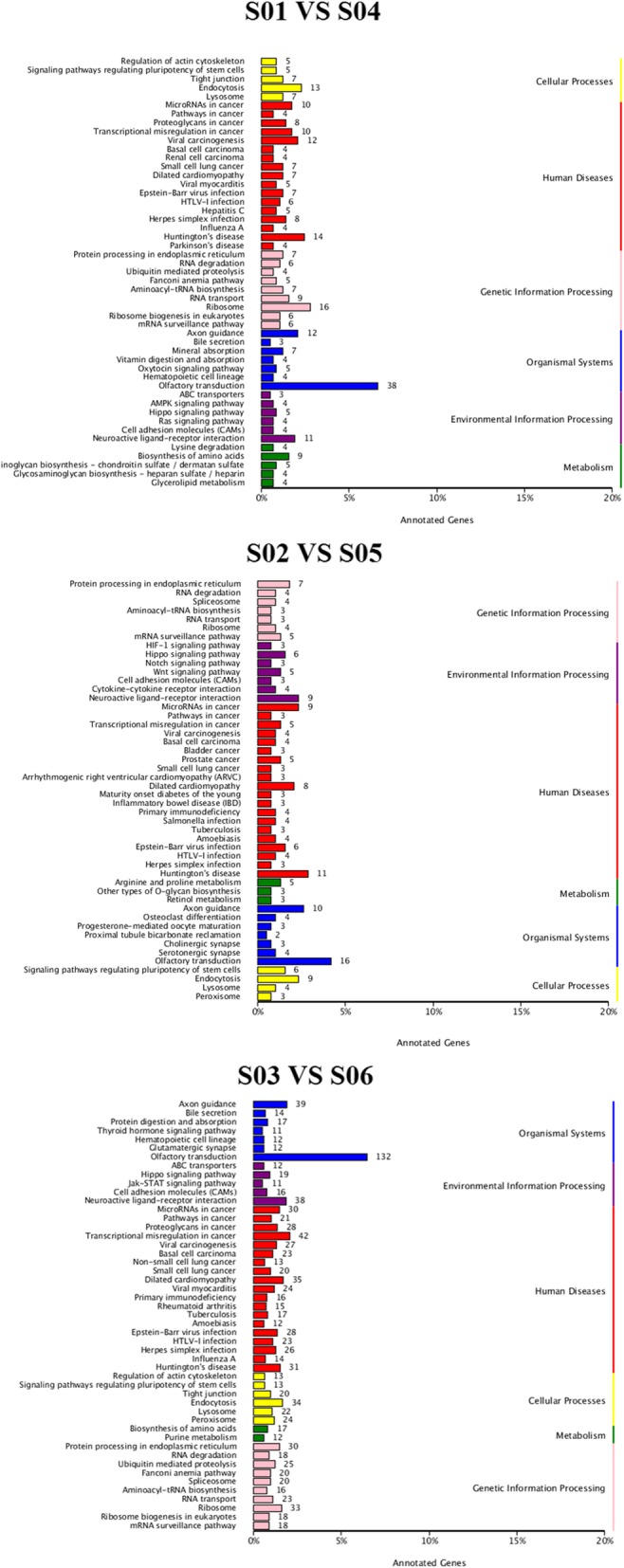
Differentially-expressed miRNA gene KEGG taxonomy. The ordinate is the name of the KEGG metabolic pathway, and the abscissa shows the number of genes annotated to this pathway and the ratio of this number to the total number of annotated genes

**Fig. 9:**
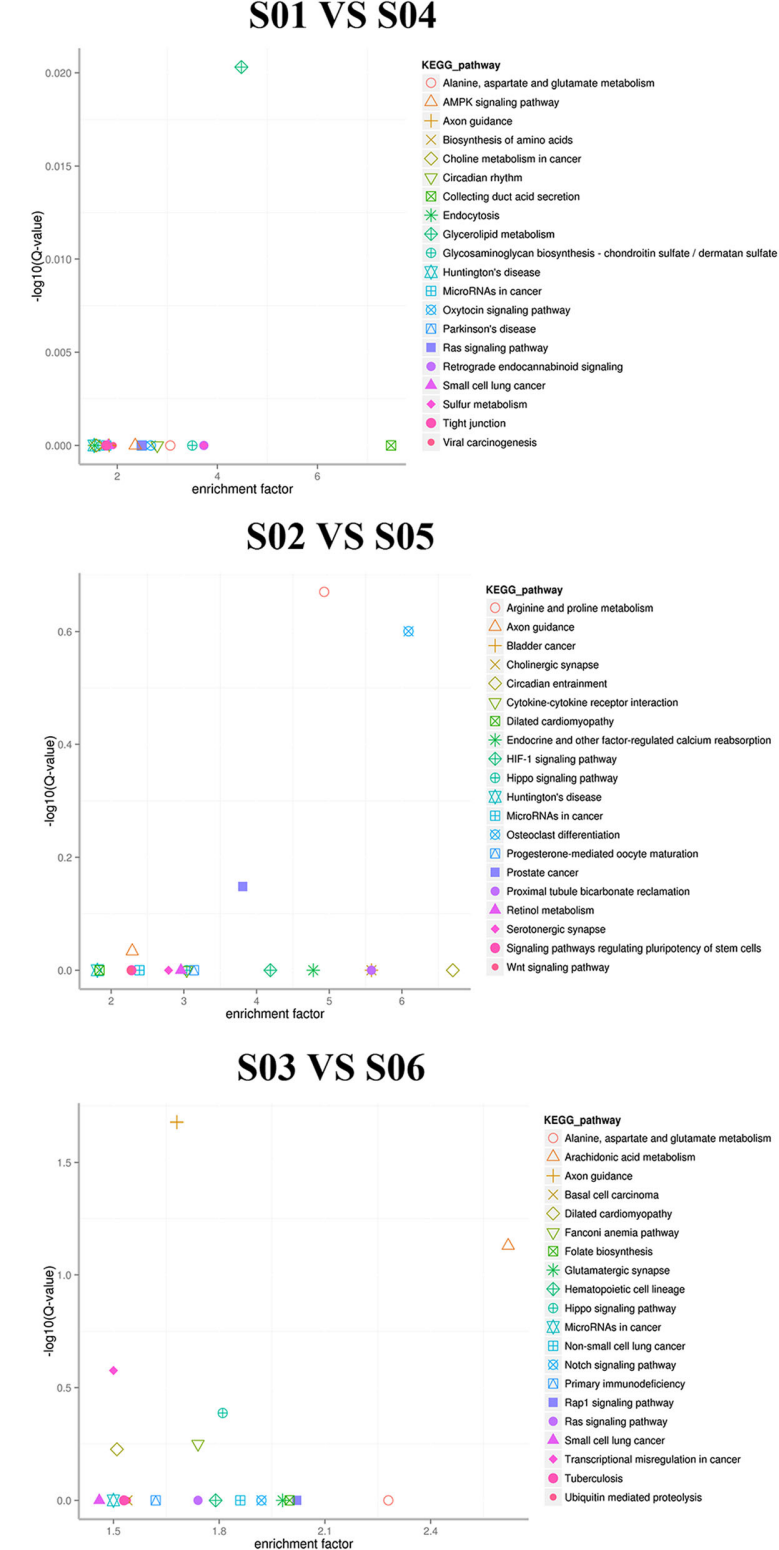
KEGG pathway enrichment scatter plot of differentially-expressed miRNA target genes. Each icon in the figure represents a KEGG pathway. The legend on the right shows pathway names. The abscissa represents the enrichment factor. The ordinate is the -log10 (Q value)

Gene Ontology (GO) annotations were determined for miRNA target genes to investigate their biological functions. The results revealed that miRNA target genes belonged to 56 functional subclasses according to the GO database. Secondary functional classes with significant differences included cell aggregation, cell killing, biological phase, biological growth, channel regulator and chemoattractants, all of which were in agreement with the results of a previous study (Fig. 7). We annotated these genes according to their KEGG pathways for statistical analysis to understand metabolic pathway involvement of the targets genes. KEGG annotation results were sorted by type of pathway (Fig. 8). An Enrichment Factor (EF) was calculated to analyze pathway enrichment, and Fisher’s method was used to assess enrichment significance (Fig. 9). Larger EF values indicated that the target genes of differentially-expressed miRNAs were significantly enriched in the pertinent pathway.

## Discussion

MicroRNAs have been linked to many biological processes, cellular components and molecular functions. Moreover, miRNAs can clearly be involved in viral infection: for instance, high expression of miR-146a can increase viral replication [23]. Many diseases can be clinically diagnosed and treated by reference to a variety of miRNA biomarkers. PRV shows differential infectivity to various host tissues. In this study, using Illumina Solexa deep sequencing technology, six small RNA libraries from the olfactory bulbs, lungs and spleens of PRV-infected and uninfected rats were constructed. Differentially-expressed miRNAs in each tissue were obtained by comparing samples from infected and uninfected animals. Differentially-expressed miRNAs with enriched expression in specific tissues were identified by comparing levels of each differentially-expressed miRNA.

### Analysis of differentially-expressed miRNAs specific to olfactory bulbs of PRV-infected rats

In mature neurons, miRNAs can affect viral evolution and regulate viral tissue tropism. Therefore, studying changes in miRNA levels during virus replication is beneficial to understanding host defense mechanisms, as well as the mechanisms that viruses use to resist them. Twenty-nine differentially-expressed miRNAs were identified by comparing olfactory bulbs of infected and uninfected rats, which included six non-conserved miRNAs and 23 conserved miRNAs. Fifteen differentially-expressed miRNAs with enriched expression in olfactory bulbs were identified, including down-regulated miRNAs (miR-1224, miR-181c-5p, miR-205, miR-298-3p, miR-412-5p, miR-873-5p) and up-regulated miRNAs (miR-26b-3p, miR-881-3p, miR-1-3p). PRV-associated differential expression of these miRNAs occurred only in the olfactory bulb. Therefore, we speculated that these miRNAs may be involved in the neurotropism of PRV. MiR-205, which was down-regulated, was predicted to be involved in regulation of neurotransmitter secretion according to its GO annotation. In Parkinson’s disease (PD), Marques et al. observed lower expression of miR-205 in brain regions of 15 PD patients, but not in brains of multiple system atrophy (MSA) patients. Thus, miR-205 level was used as a biomarker to distinguish between PD and MSA [24]. Furthermore, a low level of serum miR-205 expression was found in glioma patients [25]. In our study, miR-26b-3p was up-regulated in olfactory bulbs. According to its GO annotation, miR-26b-3p may be involved in nervous system development, spinal cord motor neuron differentiation and neural tube formation. A high level of miR-26b-3p expression also occurs in other brain diseases, such as Alzheimer’s disease. Satoh et al. showed that miR-26b-3p was expressed differentially between patient subgroups in AD using omiRas [26]. In our study, miR-873-5p was determined to be involved in the regulation of many neuronal cell biological processes according to its GO annotation, such as motor neuron axon guidance, chemorepulsion necessary for embryonic olfactory bulb interneuron precursor migration, and retinal ganglion cell axon guidance. Rats infected with PRV developed acute neurological symptoms such as trembling, twitching and muscle spasm. In addition, low levels of miR-873-5p expression were found in the hippocampus of rats in a model of temporal lobe epilepsy [27]. Thus, low levels of miR-873-5p expression may be due to neurological symptoms, which are typically associated with PRV infection.

### Analysis of differentially-expressed miRNAs specific to lungs of PRV-infected rats

PRV can cause respiratory symptoms, such as breathing difficulties and coughing. In the lungs of PRV-infected rats, 69 differentially-expressed miRNAs were identified. Forty-two differentially-expressed miRNAs were identified with enriched expression in the lung, including nine down-regulated miRNAs (miR-9a-3p, miR-653-5p, miR-488-3p, miR-361-5p, miR-329-5p, miR-129-5p, miR-128-1-5p, miR-124-3p, let-7c-1-3p), 10 up-regulated miRNAs (miR-134-5p, miR-223-3p, miR-31a-5p, miR-382-3p, miR-382-5p, miR-451-5p, miR-495, miR-505-5p, miR-6216, miR-672-3p) and 23 non-conserved miRNAs. Interestingly, miR-223-3p, miR-451-5p, miR-451-5p, miR-495, miR-505-5p, miR-361-5p, miR-129-5p, and let-7c-1-3p also showed significant differential expression in lung cancer patients. MiR-495 was up-regulated in lungs of PRV-infected rats. Researchers have found that miR-495 targeted the 3′-UTR of GRP78, leading to significant up-regulation of GRP78, especially in lung cancer. GRP78 (Gene ID: 3309) is a major regulator of the unfolded protein response (UPR) pathway. The UPR plays an important role in maintaining homeostasis within different lung cell types [28]. Studies have shown that miR-495 can target metastasis-associated protein 3 (MTA3), which is overexpressed in non-small cell lung cancer. Thus, miR-495 targeting of MTA3 may be involved in the regulation of lung cancer growth and migration [29]. In our study, miR-361-5p was minimally expressed in the lungs. As a tumor suppressor in lung cancer, miR-361-5p could directly target FOXM1 leading to down-regulation of FOXM1 expression in lung cancer cells [30]. Low expression of miR-129-5p, which could directly target DLK1, was found in the lungs of PRV-infected rats. DLK1 belongs to the epidermal growth factor-like family. Apart from its roles in regulating cell differentiation, such as osteogenesis and adipogenesis, DLK1 had been shown to contribute to tumor cell invasion and is a negative regulator of Notch signaling. Furthermore, it was shown to regulate cell stemness [31]. The let-7 miRNA family has been shown to be closely involved in drug resistance [32], carcinogenesis [33], immunocyte differentiation [34, 35] and proliferation [36]. Our study found that let-7c-1-3p was poorly expressed in lungs of PRV-infected rats. Zhao confirmed that both ITGB3 and MAP4K3 were target genes responsible for the functions of let-7c [37]. Upon let-7c overexpression, expression of ITGB3 and MAP4K3 were significantly repressed both at the protein and mRNA level. ITGB3 (also known as CD61 or integrin *β*_3_), a member of the integrin family, had been shown to be involved in “outside-in” signaling transduction and cell adhesion [38]. MAP4K3, a member of the MAP4K family, is an important component in various signaling pathways [39]. Thus, let-7c-1-3p might play a pivotal role in PRV infection and proliferation.

### Analysis of differentially-expressed miRNAs specific to spleens of PRV-infected rats

The spleen has a variety of important physiological functions in both the immune and endocrine systems. Immune function is the most important role of the spleen, both non-specifically and through its effects on T cells, B cell-mediated cellular immunity and humoral immunity. In the spleens of PRV-infected rats, 199 differentially-expressed miRNAs were identified. Moreover, 171 differentially-expressed miRNAs were identified with enriched expression in the spleen, including 102 down-regulated miRNAs and 69 up-regulated miRNAs. Many of these miRNAs were involved in regulation of immune system development, lymphocyte activation, and the immune response. The miRNA miR-10b-5p could target MAP3K7. MiR-10b overexpression inhibited production of IL-17A by both total CD4 T cells and Th17 cells [40]. MiR-125a-3p was shown to positively regulate apoptosis and differentiation in THP-1 cells. Type I interferon (IFN1) is instrumental to the cellular response against viral infections. The STAT3 signaling pathway was negatively regulated by IFN1 binding to type-I interferon receptor 1 (IFNAR1) [41, 42], and matrix metallopeptidase-9 (MMP9) is an important effector molecule in the STAT3 pathway. MiR-93-5p was identified to target IFNAR1, and MMP9 expression was increased by the miR-93-5p-IFNAR1 axis via the STAT3 pathway [43]. MiR-92b-3p directly targeted and reduced Gabra3 expression via the AKT/mTOR and JNK pathways [44]. MiR-664 directly bound to the 3′ UTR of phospholipid protein 2 (PLP2) as shown by luciferase assays, and suppressed the expression of PLP2 at the protein level as shown by western blotting. PLP2 encodes an integral membrane protein that localizes to the endoplasmic reticulum, which can multimerize and may function as an ion channel. PLP2 is necessary for down-regulation of human CD99 in the plasma membrane [45]. This result showed that CD99 expression was upregulated by reduced PLP2 levels. CD99 is a cell-surface glycoprotein involved in T-cell death, T-cell adhesion and leukocyte migration [46]. MiR-511-3p was down-regulated in the spleens of PRV-infected rats, and a recent study showed that miR-511-3p may be a potential tumor suppressor in cancer [12, 47]. Serine/threonine-specific protein kinase 3 (AKT3), which belongs to the AKT superfamily, plays an important role in cell transformation. MiR-511-3p could target the human AKT3 mRNA transcript to regulate cell transformation. MiR-486 can target KLF4 and Cezanne to jointly activate the NF-κB signaling pathway, as Cezanne and KLF4 are important negative regulators of NF-κB signaling: through different mechanisms, they can repress phospho-NF-κB-p65 (the active form of NF-κB). Cezanne, a newly identified member of the A20 family of deubiquitinases, can act as a potential tumor suppressor via NF-κB activity by deconjugating K63-polyubiquitin chains from TRAF6 and RIP. However, KLF4, a zinc-finger transcription factor, can interact with phospho-NF-κB-p65 and promote COX-2 activity [48]. All of the differentially-expressed miRNAs in the spleen were reported to be involved in immune regulation through various pathways. Thus, these differentially-expressed miRNAs may be directly involved in the antiviral immune responses of the host.

## Conclusions

We conducted a comprehensive analysis of differentially-expressed miRNAs in the olfactory bulbs, lungs and spleens of PRV-XJ infected rats and computationally predicted their putative targets. As reflected by our data, differentially-expressed miRNAs induced by PRV infection were involved in regulation of the nervous system, respiratory system and immune system, which may affect the tissue tropism of the virus as well as viral infection and proliferation. This dataset may be useful to understand the tissue tropism and mechanisms of immune escape of other herpesviruses, such as human herpes virus, at the miRNA level.

## Methods

### Animal ethics statement

All animal procedures used in the present study were conducted in accordance with good animal practices as defined by the laboratory animal use license (CertificateNo. SYXK (CHUAN) 2014–187). All work with rat was approved and supervised by the Committee on the Care and Use of Laboratory Animals of Sichuan and was conducted in the Animal Biotechnology Center of Sichuan Province at Sichuan Agricultural University. The animal interventions were performed in strict accordance with animal ethical standards. In addition, to limit any suffering, carbon dioxide (CO2) inhalation was used to anesthetize experimental animals in this study. All rats used for tissue collection were euthanized by rapid decapitation at the endpoint, which could also reduce the hemocytes in the tissues used for sequencing analysis.

### Materials

PRV-XJ strain, a wild-type PRV strain isolated in Xinjin, China, was propagated in BHK-21 cells cultured in Dulbecco’s Modified Eagle’s Medium (DMEM) supplemented with 10% (v/v) fetal bovine serum. Specific-pathogen-free male rats (180 g bodyweight) were purchased from Chengdu Dashuo Experimental Animal Co. Ltd. All animal experiments were performed in accordance with the guidelines set out by the European Convention for the Protection of Vertebrate Animals used for Experimental and other Scientific Purposes and were approved by the Medical Faculty Experimentation Ethics Committee of Sichuan Agricultural University.

### Sample collection and RNA extraction

Six rats were infected with PRV-XJ at a multiplicity of infection of 0.01 via intramuscular injection. A control group of six rats received an intramuscular injection with an equal volume of aseptic physiological saline. The infected animals were euthanized by rapid decapitation. The lungs, spleens and olfactory bulbs of rats were harvested 102 h post-infection to allow time for PRV to spread to organs and tissues, and stored in liquid nitrogen until RNA extraction. Total RNA was extracted from tissue samples using the miRNeasy Mini Kit. The miRNAs extracted from the olfactory bulbs, lungs and spleens of the six infected rats were pooled and labeled as samples PRV-Olf, PRV-Lung and PRV-Spleen, respectively. The miRNAs extracted from the olfactory bulbs, lungs and spleens of the six uninfected rats were pooled and labeled as samples Olf, Lung and Spleen, respectively.

### Library construction and sequencing

The integrity of all RNA samples was verified via Nanodrop, Qubit 2.0, and Agilent 2100 Bioanalyzer. 1.5 μg RNA per sample was used as input material to construct small RNA libraries using the small RNA Sample Prep Kit. The 3′ and 5′ ends of small RNAs were connected to adaptors using T4 RNA Ligase 1 and T4 RNA Ligase 2 (truncated), and then cDNA was synthesized using reverse transcriptase. Finally, small RNA libraries were produced using PCR, agarose gel electrophoresis, and gel extraction. Small RNA sequencing was performed using an Illumina HiSeq 2500 Genome Analyzer.

### PolyA RT-qPCR assay

Quantitation of mature miRNAs was performed as previously described. Briefly, 2 μg of total RNA was reverse transcribed into cDNA using the miRNA First Strand cDNA Synthesis Kit (Sangon Biotech) and miRNA-specific poly(A) primers. The reverse-transcription reaction was incubated at 37 °C for 60 min and at 85 °C for 5 min. To amplify miRNAs, PCR was performed a using universal reverse primer and a miRNA-specific forward primer. Real-time quantitative PCR (RT-qPCR) was performed using SYBR PremixEx Taq™ (Takara) and a Roche LightCycler® 96 (Swiss) with three replicates for testing expression differences. Triplicate reactions were performed in a total volume of 25 μL containing 12.5 μL of SYBR® Premix Ex Taq™ II (2×), 2 μL of cDNA template, 1 μL of each primer (0.5–2.5 μL), and 8.5 μL of ddH_2_O. The thermal cycling conditions were as follows: 95 °C for 1 min, 40 cycles at 95 °C for 15 s and 60 °C for 30 s. The reference rat U6 spliceosomal RNA gene, which showed only slight expression variation in RNA-seq analyses, was used for expression data normalization. Relative miRNA expression was calculated using the comparative ΔΔCt method, and values were expressed as 2^-ΔΔCt^.

### Data analysis

The raw sequence data contained adaptor sequences as well as low quality sequences. To ensure miRNA sequence accuracy, we restricted the analysis to high-quality sequences by excluding sequences with low quality, length < 18 nt, length > 30 nt as well as sequences containing ambiguous nucleotides (‘N’). Using Bowtie(v1.0.0) [49] software, the filtered reads were compared with the Silva, GtRNAdb, Rfam and Repbase databases to filter out rRNA, tRNA, snRNA, RNA (snoRNA) and other ncRNA and repeat sequences. The unannotated reads were compared with the rat reference genome (rattus_norvegicus: ftp://ftp.ensembl.org/pub/release-85/fasta/rattus_norvegicus/) to obtain positional information (mapped reads) using miRDeep2 (v2.0.5) [50] software.

### Analysis of differentially- expressed miRNAs

Previously-described and novel miRNAs were compared with sequences of reference genomes and identified using the miRDeep2 software as well as using knowledge of the biological characteristics of miRNAs(Mature sequence, Ring shaped structure, star sequence). The miRNA expression in each sample was statistically analyzed and expression levels were normalized using the TPM algorithm.

Differentially-expressed miRNAs in the lungs, spleens and olfactory bulbs of PRV-infected rats were identified by comparing miRNA sequence sets derived from infected and uninfected rats using DESeq2 software. Differentially-expressed miRNAs associated with particular tissues were identified by comparing the miRNA sequence sets in lungs, spleens and olfactory bulbs of rat regardless of infection status. MiRnada(v3.3a) and RNAhybrid(v2.1.1) software were used to predict the target genes of differentially-expressed miRNAs. The Gene Ontology (GO) program Blast2GO (https://www.blast2go.com/) was used to annotate potential host target genes and to create GO annotation histograms, including histograms for cell component, biological process and molecular function. Target genes were analyzed for their KEGG Pathway signal transduction pathway using the KEGG (http://www.kegg.jp/kegg/pathway.html) public database and compared with the NR protein database to determine the protein annotations of target genes. An Enrichment Factor (EF) was calculated to analyze pathway enrichment, and Fisher’s method was used to assess enrichment significance. Larger EF values indicated that the target genes of differentially-expressed miRNAs were significantly enriched in the pertinent pathway.

$$ \mathrm{EF}=\frac{\mathrm{The}\ \mathrm{number}\ \mathrm{of}\ \mathrm{differentially}\ \mathrm{expressed}\ \mathrm{miRNA}\ \mathrm{target}\ \mathrm{genes}\ \mathrm{in}\ \mathrm{pathway}/\mathrm{All}\ \mathrm{genes}\ \mathrm{in}\ \mathrm{pathway}}{\mathrm{The}\ \mathrm{number}\ \mathrm{of}\ \mathrm{differentially}\ \mathrm{expressed}\ \mathrm{miRNA}\ \mathrm{target}\ \mathrm{genes}\ \mathrm{in}\ \mathrm{KEGG}/\mathrm{All}\ \mathrm{genes}\ \mathrm{in}\ \mathrm{KEGG}} $$

## Data Availability

The datasets generated and/or analyzed during the current study are available in the NCBI repository (SRR6467530, SRR6467529, SRR6467532, SRR6467531, SRR6467528 and SRR6467527).

## Abbreviations

COG: Clusters of Orthologous Groups
DEGs: differentially-expressed genes
FC: Fold change
GO: Gene Ontology
IL: Interleukin
KEGG: Kyoto Encyclopedia of Genes and Genomes
LD50: Median lethal dose
NF-κB: Nuclear factor kAppa B
NLRs: NOD-like receptors
NOD: Nucleotide-binding oligomerization domain
PRV: Pseudorabies virus
SPF: Specific-pathogen-free
TLRs: Toll-like receptors
UTR: Untranslated Regions
